# A large giant cell tumor of the larynx: case report and review of the literature

**DOI:** 10.1186/s40463-017-0198-y

**Published:** 2017-04-04

**Authors:** Andrew Arndt, Rachelle LeBlanc, Peter Spafford

**Affiliations:** 1 College of Medicine, University of Saskatchewan, Saskatoon, SK Canada; 2 Clinical Professor and Division Head: Otolaryngology - Head & Neck Surgery, College of Medicine, University of Saskatchewan, Saskatoon, Saskatchewan Canada

**Keywords:** Case report, Giant cell tumor, Larynx, Thyroid cartilage

## Abstract

**Background:**

Giant cell tumors (GCTs) are typically found in the metaphyseal-epiphyseal area of long bones but can also occur in the head and neck region. GCT of the larynx is a rare entity with only 42 reported cases in the international literature. Furthermore, to the best of our knowledge this is the largest laryngeal GCT reported in the literature to date. GCT of the larynx can present with dysphonia, dyspnea, and/or dysphagia and should be considered in the differential diagnosis of a neck mass.

**Case presentation:**

This case report describes a giant cell tumor of the left thyroid cartilage in a 30-year-old man who initially presented with dysphonia and dysphagia. Computed tomography (CT) revealed a 5 × 5.7 cm mass centered on the left thyroid cartilage, which was further diagnosed by histopathology as giant cell tumour by open biopsy. The patient was counselled on treatment options and it was decided to proceed with a surgical approach. The patient consented to and successfully underwent a total laryngectomy (TL). Currently the patient has no evidence of disease at 13 months follow-up, has an optimal prosthetic voice, and is able to tolerate all textures of foods.

**Conclusion:**

GCTs of the larynx have a good prognosis and can be treated successfully through complete resection of the tumor, negating the need for adjunctive therapy such as radiation, chemo or denosumab therapy.

## Background

GCT of the cartilaginous structure of the larynx is a rare entity. To the best of our knowledge, there have only been 42 reported cases in the international literature, including Wessely’s first documented case in 1940 [1–33]. This is more than previously reported in the literature, making this condition more common than once thought, yet still extremely rare to encounter. The purpose of this case report is to contribute our clinical, pathological, imaging, and treatment outcomes associated with our patient’s laryngeal GCT and to review the current literature. Additionally, we will address the patient’s perspectives and experiences focussing on his quality of life following TL. Finally, we will comment on various treatment modalities that have been undertaken for treating laryngeal GCT’s along with their outcomes.

## Case presentation

**Fig. 1:**
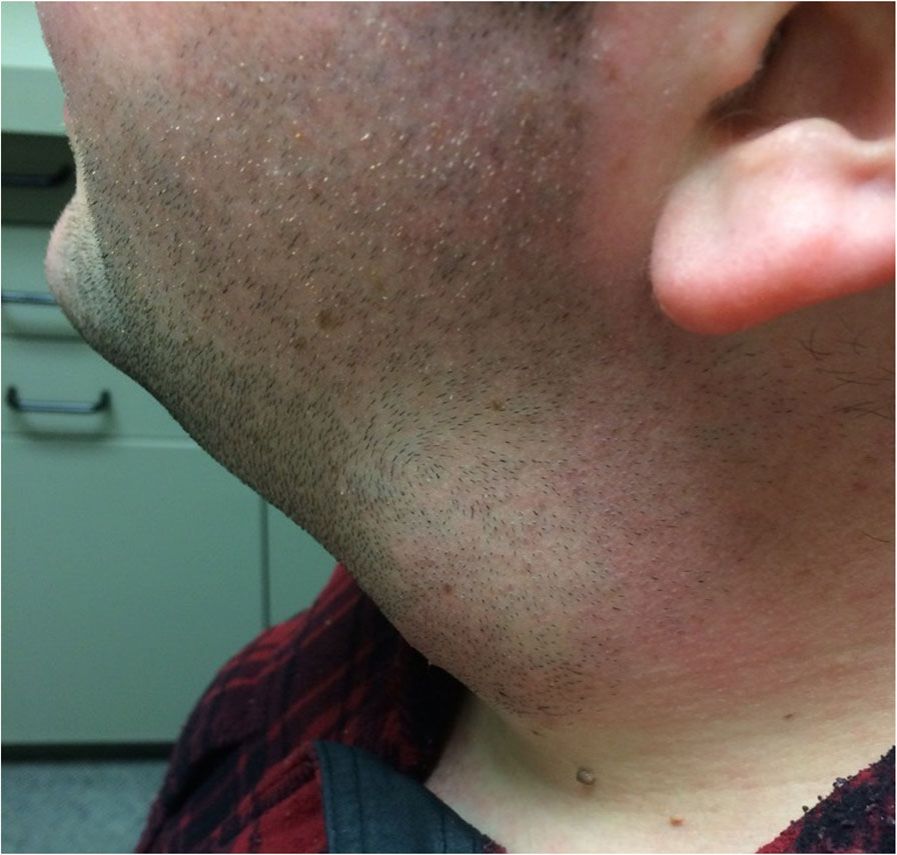
Photo of the patient revealing a left-sided neck mass

A 31-year-old Bosnian male with an unremarkable health history was originally seen in early October 2015 at a walk-in medical clinic after he noticed a change in his voice for 3 weeks. The patient had no smoking history, no prior occupational exposures, was an infrequent social drinker and worked as an assistant manager at a convenience store. Initially he thought the hoarseness was secondary to an upper respiratory tract infection and surprisingly did not notice the neck mass until it was pointed out to him (Fig. 1). The physician who examined him at the time felt a large mass in the left anterior portion of the neck and followed up with a neck ultrasound. The neck ultrasound was unremarkable revealing a homogeneous thyroid gland with no solid or cystic soft tissue masses along with normal appearing lymph nodes on both sides of the neck.

**Fig. 2:**
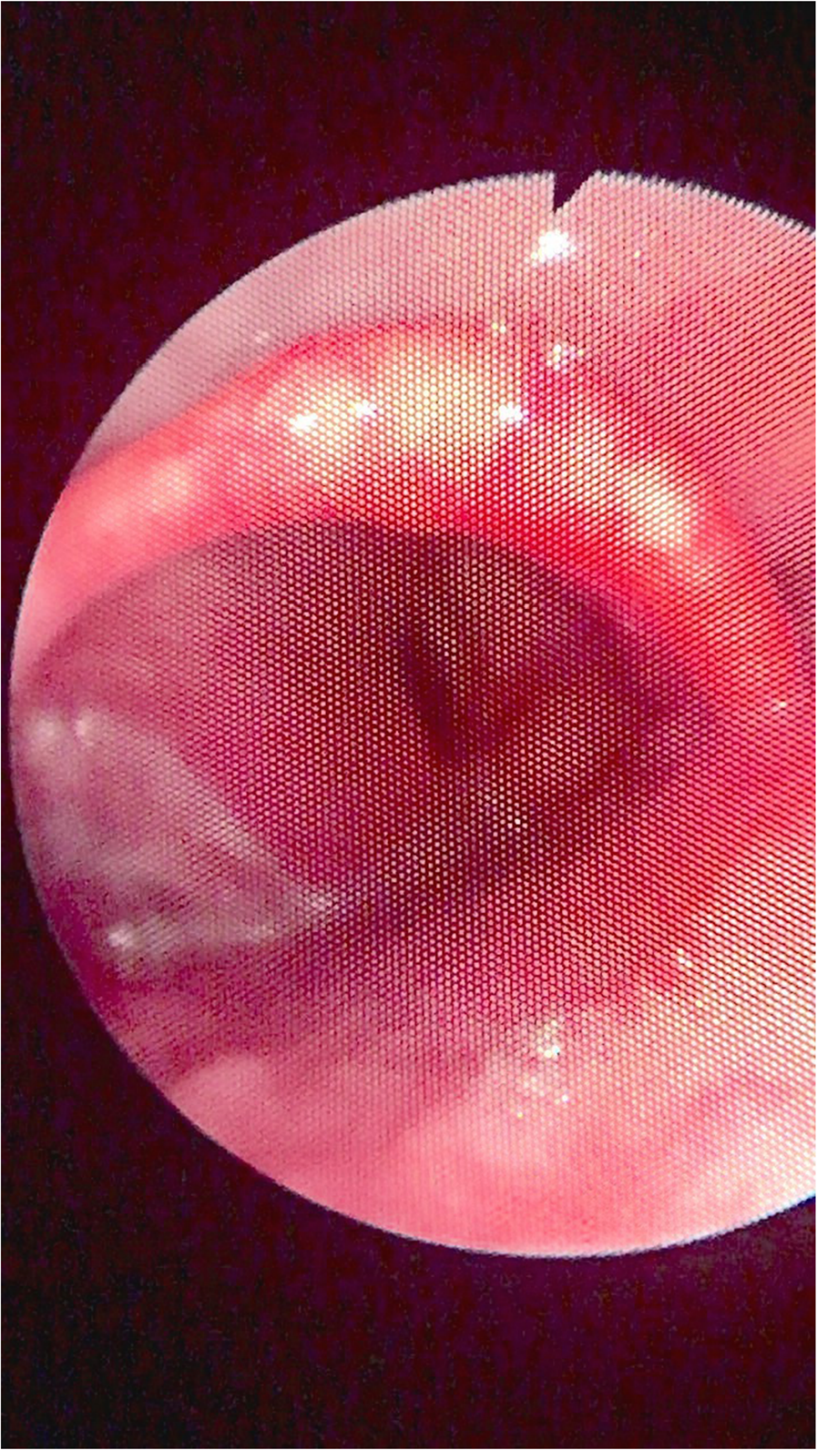
Direct nasopharyngolaryngoscopy showing a left submucosal mass involving the left vocal cord

Two days later he presented to the emergency department because of increasing hoarseness and mild dysphagia. The department of Otolaryngology Head and Neck Surgery was consulted. At the time of consultation, the patient appeared to be in no airway distress and had normal vital signs. On physical examination, a palpable mass was felt on the left side of the neck. The rest of the head and neck examination was unremarkable. Laboratory investigations were ordered and included CBC, serum ionized calcium, thyroid stimulating hormone, free T4 and T3 and calcitonin. These laboratory findings were all within normal limits with the exception of a slightly elevated T3 level. Direct fiberoptic nasopharyngolaryngoscopy was performed and revealed a supraglottic mass involving the left vocal cord causing immobility (Fig. 2). The mass appeared to be submucosal in nature and did not affect the right vocal cord. He was then admitted for observation and further workup for diagnostic purposes.

**Fig. 3:**
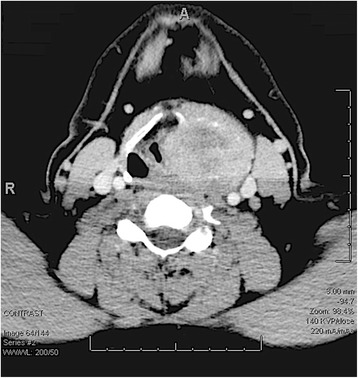
CT with contrast of the neck showing a large laryngeal mass destroying the left thyroid cartilage

A CT scan with contrast was performed and showed a 5 × 5.7 cm laryngeal mass centered on the left thyroid cartilage, which was completely destroyed (Fig. 3). It displayed some central decreased attenuation and was associated with early punctate calcification. The tumor extended medially and compressed the laryngeal ventricle and airway at the level of the vocal cords. Superiorly it extended to the level of the left intact hyoid bone and compressed the left pyriform sinus. There was compression and displacement of the left carotid sheath and left sternocleidomastoid with no sign of any direct invasion. A 1.1 cm diameter lymph node was seen just lateral to the jugular vein and inferior to the tumor on the left-hand side. No other bone or soft tissue abnormality was observed. At this point the appearance of the mass was very suggestive of a chondrosarcoma. Magnetic resonance imaging (MRI) revealed the same findings as the CT scan and maintained the suspicion for a chondrosarcoma.

**Fig. 4:**
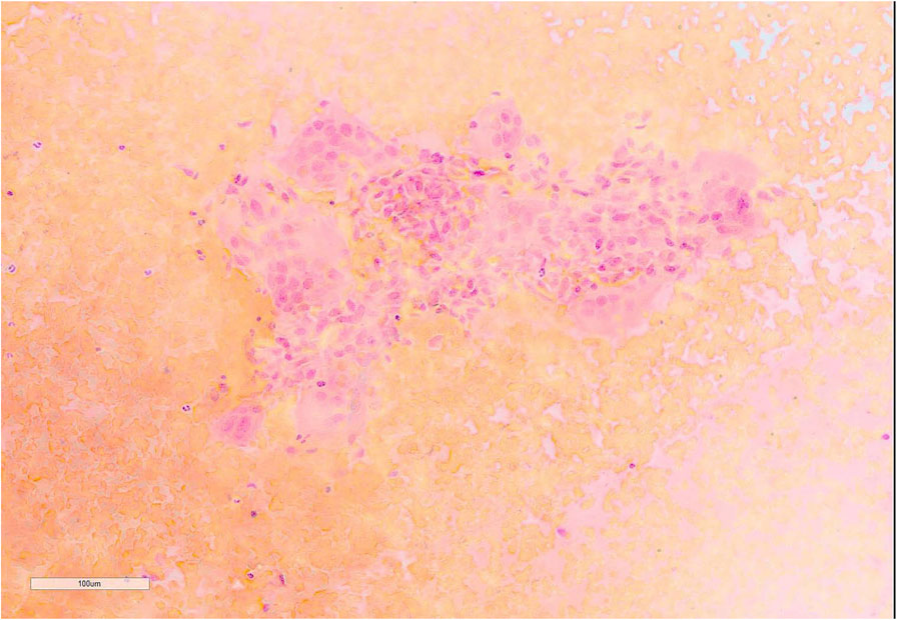
Papanicolaou staining in high power showing tight groups of spindle and epithelioid cells wrapped around prominent vessels. Multiple giant cells are also noted

**Fig. 5:**
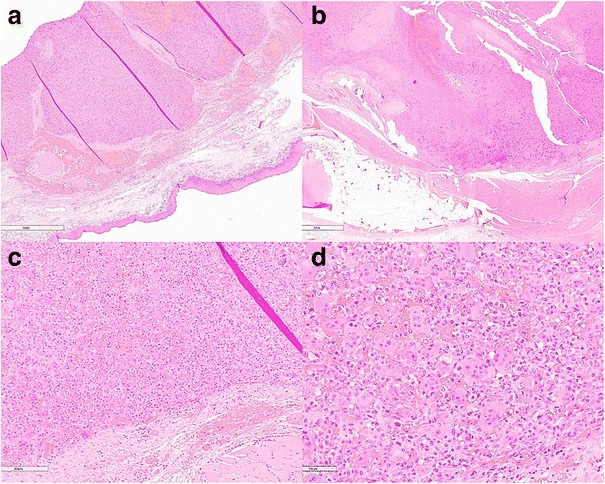
Hematoxylin and eosin staining: **a**) Low power of squamous mucosa with underlying submucosal tumor **b**) Low power showing relationship of thyroid cartilage and tumor. **c** Tumor with vascular invasion **d**) High power showing prominent giant cells with spindled cells and small vessels between

To clarify the diagnosis, an open biopsy was performed. Histopathological findings from the biopsy specimen revealed uniform sheet appearance of multinucleated osteoclast-like giant cells (Figs. 4 and 5a-d). The intervening cells between the giant cells showed band nuclear features supporting a diagnosis of giant cell tumor.

The patient’s case was discussed at the Saskatoon Cancer Centre Head and Neck rounds and the consensus was to proceed with a surgical approach. After consulting with various head and neck cancer specialists across Canada, reviewing the literature, and respecting the patient’s wishes it was decided to proceed with a TL. The decision to proceed with a TL versus a PL was extremely difficult. With the extreme size of the tumor and significant pharyngeal invasion, it was postulated that a partial laryngectomy in this particular case, would result in a high degree of remaining laryngeal dysfunction.

**Fig. 6:**
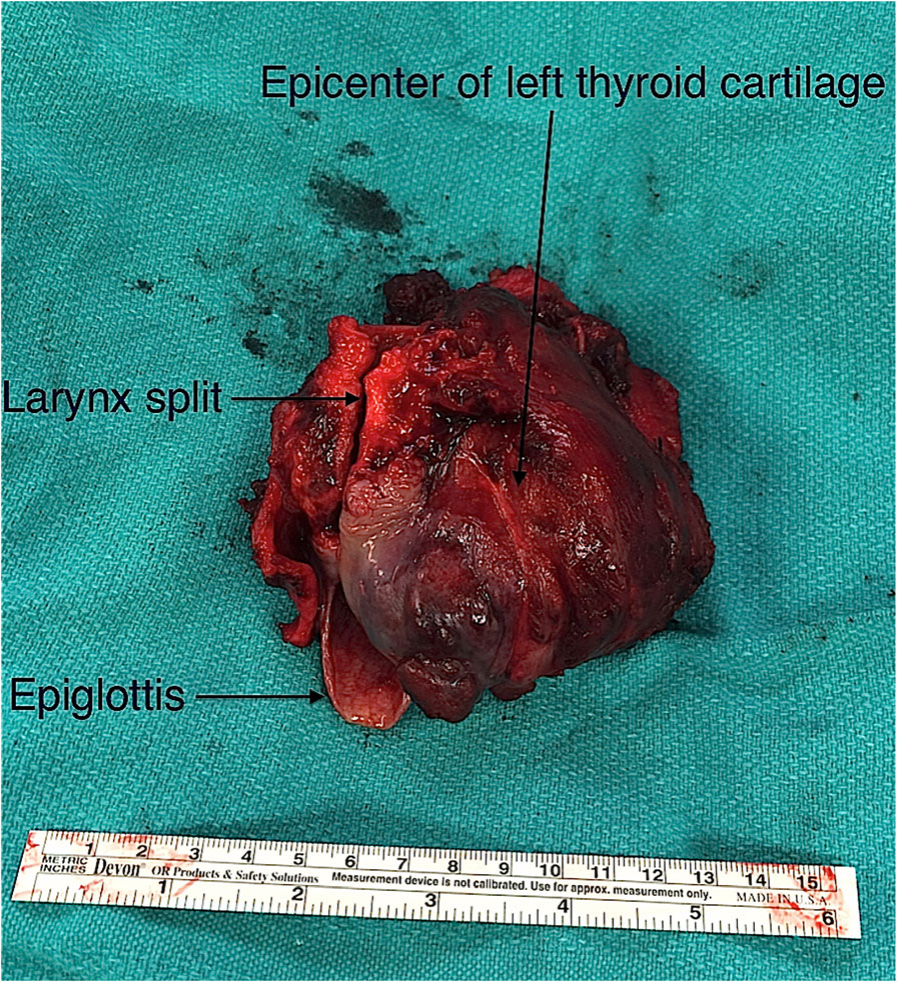
Gross specimen. The tumour starts superior at the base of the epiglottis with destruction of the left thyroid cartilage and extends inferiorly to the first tracheal ring

On December 2015, the patient underwent surgery. The following procedures were performed: total laryngectomy, left hemithyroidectomy, pharyngeal plexus neurectomy, partial pharyngectomy, anterior pharyngotomy, and creation of tracheoesophageal fistula for future voice prosthesis (Fig. 6).

At 13 months follow-up the patient has no evidence of disease or recurrence. He is able to tolerate all textures of foods and is happy with the quality of his voice. In terms of communication he states he “doesn’t have any problems” and has been doing “very well from a psychological standpoint”. He was connected with an existing laryngectomy patient for peer support prior to surgery, which he found very helpful and gave him “a piece of mind.”

He currently works two jobs, the first being an assistant manager at a convenience store and second as an office clerk. He has an interdisciplinary degree in political science and business administration and is hoping to return to school in the future. One day he hopes to swim again as this was his main form of exercise and stress relief prior to surgery. The patient will have continued follow up in clinic to ensure no tumor recurrence.

## Discussion

GCTs are typically found in the epiphyseal area of long bones and have a predilection for females in their third decade [1]. These tumors are usually benign in nature but can be locally aggressive, recur, and occasionally undergo pulmonary metastasis [1, 31]. About 4% of all primary bone tumors are GCTs, with approximately 2% of all GCTs occurring in the head and neck region [22]. Most often GCTs in the head neck region occur in the sphenoid, ethmoid, or temporal bones and very rarely in the hyoid bone and cartilaginous framework of the larynx [22, 34]. Wessely reported the first case of a laryngeal GCT in 1940 [1]. Including our case and based on our literature search, there have been an additional 42 reported cases (Table 1) [1–33].

**Table 1:** Previously reported cases of giant cell tumor of the larynx

Case	Year	Author	Age	Sex	Location	Size (cm)	Treatment
1	1940	Wessely [1]	51	Male	Cricoid		RT
2	1951	Federova [2]	35	Male	Thyroid		PL + RT
3	1952	Wagemann [3]	40	Male	Cricoid		TL + RT
4	1958	Perrino [4]	32	Male	Cricoid		TE via LF + RT
5	1966	Kaliteevskii and Korol’kova [5]	52	Male	Thyroid	5	TL
6	1968	Pohl [6]	50	Male	Thyroid		RT
7	1969	Kohn [1]	50	Male	Epiglottis		
8	1971	Rudert [7]	53	Male	Thyroid		PL
9	1972	Hall-Jones [8]	26	Male	Thyroid	5.5	TL
10	1973	Goto and Nakashima [9]	47	Male	Thyroid		TE
11	1974	Kotarba and Niezabitowski [10]	60	Male	Epiglottis	3	PL
12	1975	Ribari et al. [11]	35	Male	Cricoid	“Walnut”	TE via LF + RT
13	1976	Kubo et al. [12]	40	Male	Cricoid	2.2	TE via LF + RT + 5 FU
14	1988	Borghese et al. [1]	28	Male	Thyroid		TE via LF
15	1992	Badet et al. [13]	55	Female	Cricoid		TL
16	1993	Murrell and Lantz [14]	42	Male	Thyroid	4	TL
17	1994	Martin et al. [15]	23	Male	Thyroid	4	PL
18	1994	Miyata et al. [16]	60	Male	Thyroid	3	TL
19	1997	Werner et al. [17]	35	Male	Thyroid	4.8	TL
20	1997	Paik et al. [18]	39	Male	Thyroid	5	TL
21	2000	Hinni [19]	31	Male	Thyroid	4	PL
22	2001	Wieneke et al. [20]	37	Male	Thyroid	4.2^a^	RT + Cytoxan + TL
23	2001	Wieneke et al. [20]	37	Male	Cricoid	4.2^a^	PL
24	2001	Wieneke et al. [20]	40	Male	Thyroid	4.2^a^	PL
25	2001	Wieneke et al. [20]	44	Male	Thyroid	4.2^a^	TL
26	2001	Wieneke et al. [20]	53	Female	Thyroid	4.2^a^	PL
27	2001	Wieneke et al. [20]	57	Male	Cricoid	4.2^a^	RT
28	2001	Wieneke et al. [20]	62	Female	Thyroid	4.2^a^	TL
29	2004	Wong and Seikaly [21]	49	Male	Thyroid	4	PL
30	2007	Nishimura et al. [22]	31	Male	Thyroid	4	PL
31	2007	Chang et al. [23]	53	Male	Thyroid		
32	2008	Zheng-Ping et al. [24]	53	Male	Thyroid	3	PL
33	2012	Le et al. [25]	39	Male	Thyroid	5	PL
34	2013	Derbel et al. [26]	38	Male	Thyroid	4	Denosumab + PL
35	2013	Lv et al. [27]	65	Male	Thyroid	4	PL
36	2013	Vivero et al. [28]	34	Male	Thyroid	4.5	PL + Chemotherapy
37	2014	Chunling et al. [29]	39	Male	Thyroid	3.6	TE
38	2014	Leon-Medina et al. [30]	40	Male	Thyroid	1.5	TL
39	2014	Nota et al. [31]	59	Male	Thyroid	3	TL
40	2015	Swanson and Brown [32]	76	Female	Cricoid	1	TE
41	2015	Yancoskie et al. [1]	46	Male	Thyroid	4.5	PL + Denosumab
42	2016	Lida et al. [33]	53	Male	Thyroid	4.6	TL
43	2016	Present case	31	Male	Thyroid	5.7	TL

*NED* no evidence of disease, *TL* total laryngectomy, *PL* partial laryngectomy, *TE* tumor excision, *LF* laryngofissure, *RT* radiation therapy, *FU* fluorouracil, ^a^ Size averaged by author

Our literature search included all cases of true GCT arising from the laryngeal skeleton. We excluded cases that arose externally to the laryngeal skeleton or cases that were questionable in regards to the origin of the tumor. Of note, we excluded four separate cases that have been previously reported as primary GCTs of the larynx. Tsybyrne concluded that based on clinical, radiological and cytological findings the final diagnosis as a low-grade thyroid cancer with spread to the thyroid cartilage [35]. Coyas et al*.* and Froboese et al*.* both reported cases of GCT of the vocal cord but without involvement of the laryngeal skeleton [36, 37]. These two cases would now be classified as GCT of the soft tissue [38]. Lastly, Stepanov et al*.* reported a case of laryngeal GCT that had metastasized from the femur [39].

Based on our case and review of the literature, laryngeal GCTs occur more frequently in males (*n* = 39) than females (*n* = 4) and have an average age at presentation of 44.6 years [1–33]. The most common site affected is the thyroid cartilage (*n* = 32) followed by cricoid cartilage (*n* = 9), and epiglottis (*n* = 2). The size of the tumors were measured by either gross examination or medical imaging. The average tumor size for the reported cases is 3.9 cm. The most common treatment is surgical resection (*n* = 29) followed by surgery and radiation therapy (*n* = 4), radiation therapy alone (*n* = 3), surgery with chemo and radiation therapy (*n* = 2), surgery plus denosumab (*n* = 2) and surgery with chemo (*n* = 1). The average follow-up period for those reported is 4.3 years. All cases that reported follow-up had no evidence of disease with the exception of Swanson and Brown, who reported observing a small subepiglottic nodule at 0.8 years follow up [32]. However, the patient is asymptomatic and is currently being observed. Lastly, only one case was reported as a GCT with osteosarcomatous malignant transformation [28]. The patient successfully underwent a PL with adjuvant chemotherapy and was free of recurrence at 6.3 years follow-up [28].

A large tumour appearing over the thyroid cartilage area has a large differential diagnosis, in which a GCT should be included. The differential diagnosis includes but is not limited to: giant cell reparative granuloma, brown tumor of hyperparathyroidism, osteoblastoma, chondroblastoma, chondrosarcoma, chondroma, aneurysmal bone cysts, nonossifying fibroma, foreign body reaction, benign fibrous histiocytoma, malignant fibrous histiocytoma, osteosarcoma with abundant giant cells, and spindle cell or sarcomatoid carcinoma with giant cells [25]. Given the uncertainty of such a tumor clinically and the difficulty of distinguishing GCT from the above listed lesions radiographically, it is imperative to make the distinction through histological examination [25]. This will allow for a definitive diagnosis and will assist on how to proceed next in regards to treatment.

Recently there has been some evidence supporting the use of systemic denosumab (a monoclonal antibody against the RANK ligand) as a treatment option for GCT of the bone [40]. However, its long-term safety and efficacy for laryngeal GCTs is unknown as only 2 reported cases have been treated with denosumab. Derbel et al. observed a reduction of the tumor volume (12.4 to 10.35 ml) along with complete histological response showing absence of giant cells after 3 months of systemic denosumab [26]. However, a PL was still performed as a definitive treatment [26]. Yancoskie et al. initiated denosumab therapy with their patient for 2 months following a PL with no signs of recurrence at 8 months follow-up [1].

At present the literature supports surgical management of laryngeal GCTs. However, there is an evolving role for novel therapeutics such as denosumab to potentially minimize the morbidity of treatment. Factors that will influence the extent of surgery (TL vs. PL) include: 1) potential recurrence 2) post-operative function and quality of life and 3) if one does choose to do a PL, then management would more likely include ancillary measures such as radiation therapy, chemotherapy, or additional salvage surgery. Furthermore, given the fact the average age at presentation is 44.6 years gives all the more reason to avoid radiation therapy as sarcomatous transformation is of concern [24].

## Conclusion

GCTs of the larynx are benign tumors and have a seemingly high cure rate. The main management dilemma is focused upon the extent of laryngeal surgery i.e. laryngeal preservation. There is paucity in the literature related to functional outcomes and quality of life as factors in deciding upon management. With respect to this, surgeons need to be more diligent in reporting follow-up relating to laryngeal function and patient quality of life. With patient counselling and consent, a TL is an effective treatment for an extremely large laryngeal GCT with significant invasion of surrounding tissue. Thus fulfilling the goals of guaranteeing a low recurrence rate and negating the need for adjunctive therapy such as radiation, chemo, or denosumab therapy and their potential complications.

## Abbreviations

CBC: Complete blood count
CT: Computed tomography
GTC: Giant cell tumor
PL: Partial laryngectomy
RANK: Receptor activator of nuclear factor kappa-B
T3: Triiodothyronine
T4: Thyroxine
TL: Total laryngectomy
